# Near-Infrared Spectroscopy Assessment of Tissue Oxygenation During Selective Cerebral Perfusion for Neonatal Aortic Arch Reconstruction

**DOI:** 10.3389/fmed.2021.637257

**Published:** 2021-04-30

**Authors:** Chi-Hsiang Huang, Yi-Chia Wang, Hen-Wen Chou, Shu-Chien Huang

**Affiliations:** ^1^Department of Anesthesiology, National Taiwan University Hospital, Taipei, Taiwan; ^2^Department of Surgery, National Taiwan University Hospital, Taipei, Taiwan

**Keywords:** selective cerebral perfusion, cardiopulmonary bypass, hypothermia, near-infrared tissue saturation, aortic arch surgery

## Abstract

**Objective:** Optimal selective cerebral perfusion (SCP) management for neonatal aortic arch surgery has not been extensively studied. We induced mild hypothermia during SCP and used the tissue oxygenation monitor to ensure adequate perfusion during the cardiopulmonary bypass (CPB).

**Methods:** Eight cases were recruited from September 2018 to April 2020. SCP was maintained at 30°C, and CPB was adjusted to achieve a mean right radial artery pressure of 30 mmHg. The near-infrared tissue saturation (NIRS) monitor was applied to assess the right and left brain, flank, and lower extremity during the surgery.

**Results:** During surgery, the mean age was 4.75 days, the mean body weight was 2.92 kg, the CPB duration was 86.5 ±18.7 min, the aortic cross-clamp time was 46.1 ± 12.7 min, and the SCP duration was 14.6±3.4 min. The brain NIRS before, during, and after SCP was 64.2, 67.2, and 71.5 on the left side and 67.9, 66.2, and 70.1 on the right side (*p* = NS), respectively. However, renal and lower extremity tissue oxygenation, respectively decreased from 61.6 and 62.4 before SCP to 37.7 and 39.9 after SCP (*p* < 0.05) and then increased to 70.1 and 90.4 after full body flow resumed. No stroke was reported postoperatively.

**Conclusion:** SCP under mild hypothermia can aid in efficient maintenance of brain perfusion during neonatal arch reconstruction. The clinical outcome of this strategy was favorable for up to 20 min, but the safety duration of lower body ischemia warrants further analysis.

## Introduction

The optimal perfusion strategy for infant aortic arch reconstruction surgery is debatable. Deep hypothermia can reduce the metabolic demand and prolong the ischemic tolerance, and circulatory arrest can provide a bloodless surgical field during aortic reconstruction; therefore, deep hypothermic circulatory arrest (DHCA) has been used in mainstream cardiopulmonary bypass (CPB) management for aortic arch reconstruction. However, many lines of evidence have indicated its deleterious effect on the brain (1). The safe threshold for DHCA duration remains uncertain. Selective cerebral perfusion (SCP) has been adopted as an adjunct or alternative method for arch repair surgery (2). Antegrade cerebral perfusion through the innominate artery can enable symmetric blood flow in neonates with a normal circle of Willis (3). Comparable perioperative and short-term neurological outcomes have been achieved using both SCP and DHCA in complex neonatal arch reconstruction surgical procedures (4). However, visceral organ perfusion is not achieved in SCP; thus, the impact of the distal ischemia remains controversial (5, 6). The Society of Thoracic Surgeons Congenital Heart Surgery Database indicated that SCP is selected in 59% of the surgical procedures as either the main perfusion strategy or a strategy in combination with DHCA. DHCA is still used in a substantial percentage of patients (7).

Studies on aortic arch surgery and SCP in neonates differ in terms of patient groups and surgical management (4, 8, 9). In adult elective aortic arch surgery, moderate hypothermic circulatory arrest with selective antegrade cerebral perfusion can be a safe procedure in terms of visceral organ complications, neurocognitive morbidity, and mortality, but the relevant evidence in neonates is limited (5). Many centers perform neonatal complex aortic arch surgical procedure by employing SCP under deep hypothermia at 18–22°C (10–12). In recent years, mild hypothermia has become an increasingly popular choice for neonatal cardiac surgery instead of deep hypothermia. However, whether SCP provides the most favorable postoperative outcomes in short procedure under mild hypothermia was unknown (6). Because the optimal temperature for SCP has not been determined, we conducted a prospective observational study on neonate aortic arch reconstruction with mild hypothermia and SCP.

## Materials and Methods

### Study Design

After obtaining approval from the institutional ethics committee, we conducted this single-institutional (tertiary university-affiliated medical center) cohort study to analyze outcomes of neonates undergoing aortic arch surgery from September 2018 to April 2020. Patients who were in profound shock and with abnormalities other than cardiac defects were excluded. Data such as age, sex, gestational age, body weight, body height, diagnosis, and preoperative inotropic support were recorded. The vasoactive-inotropic score was measured as follows: dopamine dose (μg/kg/min) + dobutamine dose (μg/kg/min) + 100 × epinephrine dose (μg/kg/min) + 10 × milrinone dose (μg/kg/min) + 10,000 × vasopressin dose (U/kg/min) + 100 × norepinephrine dose (μg/kg/min) (13). The following intraoperative variables were obtained: duration of CPB, duration of hypothermic circulatory arrest, minimum hematocrit during CPB, lowest rectal temperature attained during CPB, duration of peritoneal dialysis, ventilator use, urine output, and hospital stay. The follow-up continued until October 2020.

### Surgical Technique

**Figure 1:**
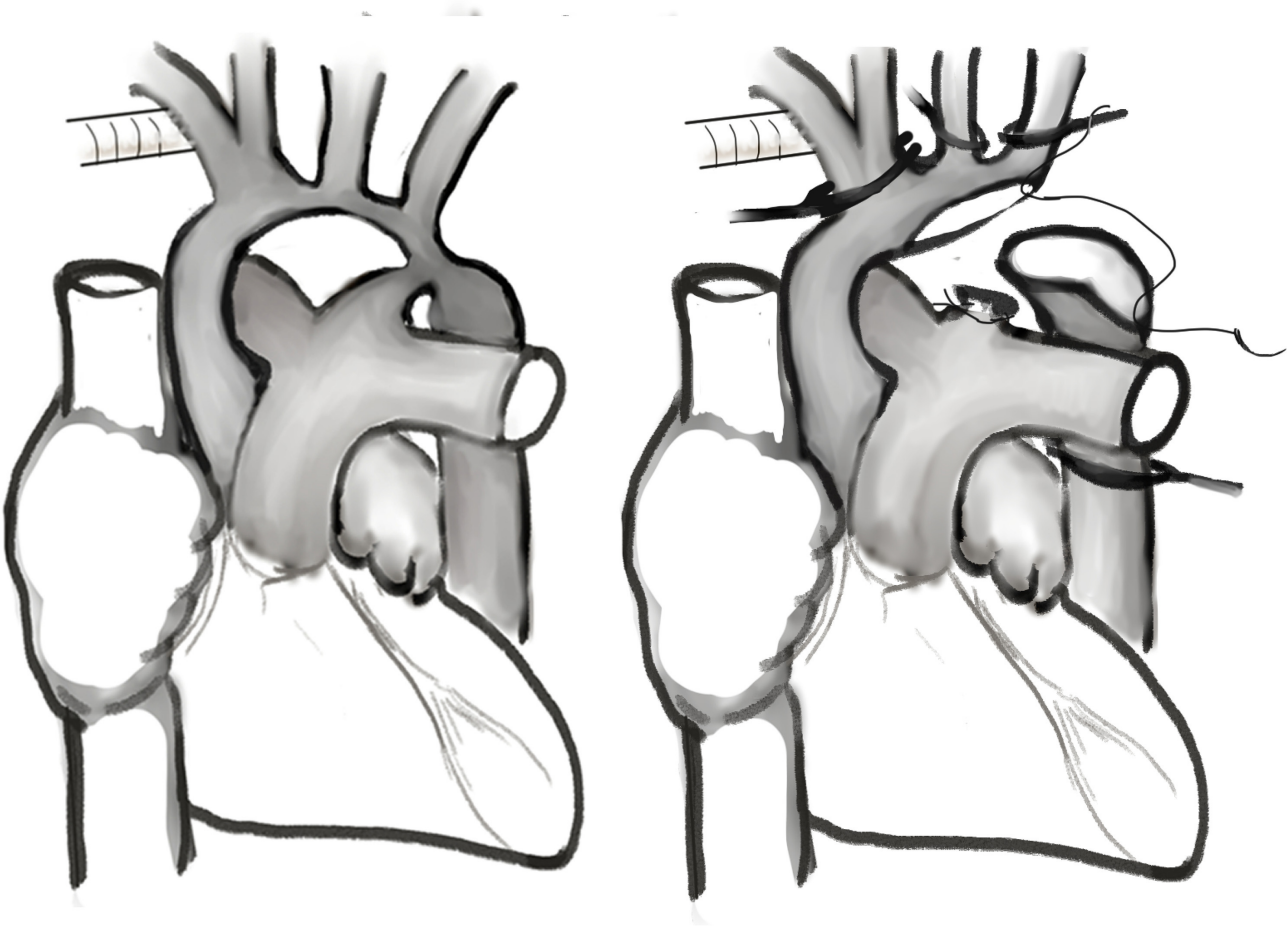
Neonatal aortic arch repair with selective cerebral perfusion.

As shown in Figure 1, an arterial cannula was inserted into a 3.5-mm expanded polytetrafluoroethylene tube (Gore-Tex; W.L.Gore & Associates, Flagstaff, AZ, USA) that was anastomosed to the innominate artery. Another arterial cannula was inserted into the descending aorta through the ductus arteriosus for lower body perfusion during the cooling phase. After insertion of a venous cannula into the right atrium, cardiopulmonary bypass was conducted at a flow rate of 150–180 mL/kg/min. During the cooling phase, the ductus arteriosus was ligated and divided from the pulmonary artery (proximal to the cannulation site). The descending thoracic aorta was extensively mobilized through blunt dissection as far distally as possible for further reconstruction surgery (14). After the target temperature was achieved, the cannula through the descending aorta was removed, the innominate artery was clamped, and SCP was started. Arch reconstruction is typically performed through resection of the coarctation tissue and extended end-to-end anastomosis. A peritoneal dialysis catheter was inserted during the operation to augment fluid removal in selective patients (15).

### Perfusion Methods

Blood gas management was performed using the alpha stat technique. Regional cerebral perfusion rates were maintained between 35 and 40 mL/kg/min with a rectal temperature of 30°C and the right radial artery pressure between 30 and 40 mmHg.

### Near-Infrared Spectroscopy Measurements

During the perioperative period, regional saturations of the bilateral frontal forehead, left flank, and left thigh were estimated using near-infrared spectroscopy (NIRS) with neonatal cerebral and somatic sensors (INVOS 5100C; COVIDIEN, Mansfield, MA, USA). The left kidney position was confirmed using ultrasound before flank somatic sensor placement.

### Statistical Analysis

Descriptive data for continuous variables are shown as means and their standard deviations (SDs). Differences in continuous variables before and during SCP were compared using the Student *t*-test. GraphPad Prism (version 6.0; GraphPad Software, La Jolla, CA, USA) was used for statistical analysis and generation of graphics.

## Results

Among the eight patients who participated in the study, five had coarctation of the aorta (CoA) with ventricular septal defect (VSD), and three had interrupted aortic arch type A with VSD and atrial septal defect. One patient with CoA with VSD underwent DHCA during arch repair surgery because during the right innominate artery cannulation, clamping of the innominate artery caused simultaneous blood pressure drop in the lower extremity. The other seven patients underwent surgery with SCP for arch repair. The mean body weight of the patients was 2.93 kg. Their preoperative data are shown in the Supplementary Table 1. The mean cardiopulmonary bypass duration was 86.5 ± 18.7 min, the aortic cross-clamp time was 46.1 ± 12.7 min, and the SCP duration was 14.6 ± 3.4 min. The cardiopulmonary bypass setting and duration are shown in Table 1.

**Table 1:** Cardiopulmonary bypass setting for included patients.

	**Operation duration (min)**	**Aortic cross-clamp duration (min)**	**Total CPB duration (min)**	**SCP duration (min)**	**SCP Temperature (°C)**	**SCP MBP (mmHg)**
1	129	45	75	13	33.1	25.7
2	139	39	83	14	29.1	32
3	152	58	93	20	29.6	28
4	153	56	91	14	31	27
5	167	50	82	18	29.6	27
6	137	22	55	9	32	31.5
7	157	39	92	13	32.8	26.7
8	160	60	121	16	20.8	6.7

*CPB, cardiopulmonary bypass; SCP, selective cerebral perfusion; MBP, mean blood pressure*.

**Figure 2:**
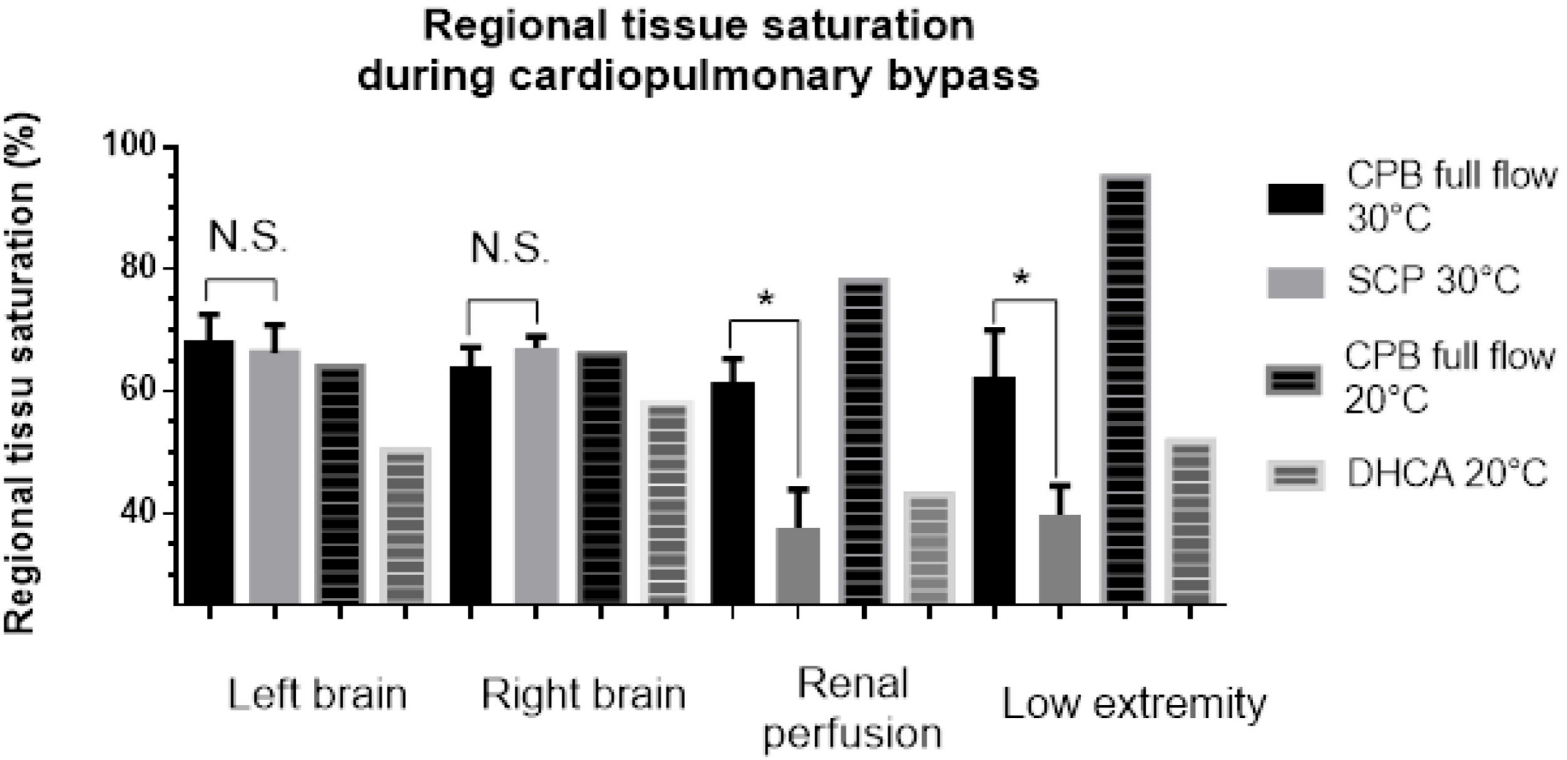
Tissue saturation in different organs in selective cerebral perfusion patients under mild hypothermia and deep hypothermic cardiac arrest patients.

The regional saturation in the bilateral frontal area did not differ between the SCP period and the full-flow period (right side full flow vs. SCP: 67.9 ± 4.7% vs. 66.2 ± 4.6%; *p* = 0.81; left side full flow vs. SCP: 64.2 ± 3.0% vs. 67.2 ± 1.8%; *p* = 0.42). The regional saturation in the left flank area was significantly lower during SCP (61.6 ± 3.7% vs. 37.7 ± 6.2%; *p* = 0.01). The regional saturation in the left thigh area was significantly lower during SCP (62.4 ± 7.56% vs. 39.9 ± 4.71%; *p* = 0.02; Figure 2). In patients who underwent DHCA, the cerebral regional saturation decreased by 21 and 44%, respectively, in the left and right brain during total circulatory arrest status. The saturation levels in the flank and lower extremity were high at low temperature with full flow and decreased during SCP and lower body circulatory arrest. We compared the absolute regional saturation data in SCP patients at 30°C and DHCA patients at 20°C and found that the regional saturation for the DHCA patients was 15% higher in the flank and 30% higher in the lower extremity compared with the SCP patients.

No death, stroke, or seizure events were reported in our patients. As shown in Table 2, two patients who had SCP had inadequate urine output in the postoperative day 1, but their urine output increased to more than 0.5 mL/kg/h in postoperative day 2. A peritoneal dialysis catheter was inserted perioperatively and removed uneventfully for 7 and 5 days in postoperative care. The patients who underwent DHCA needed the longest ventilator support in the intensive care unit. All the patients recovered favorably and were followed up regularly at the outpatient clinic.

**Table 2:** Clinical outcomes for included patients.

	**Post-operation ECMO**	**ICU stay (day)**	**Peritoneal dialysis (day)**	**Urine output by mL/kg/h in post-operative day 1 (mL)**	**Lactate immediately after CPB (mmole/L)**	**Post-operation day 1 lactate (mmole/L)**	**VIS before ICU admission**
1	0	2	0	5.46	3.4	2.49	6.3
2	0	2	0	5.17	4.01	3.65	5
3	0	3	0	0.37	3.89	3.03	9
4	0	3	0	0.65	7.05	5.08	8.6
5	0	9	7	0.51	5.46	4.16	16
6	0	3	0	5.40	4.69	3.37	11
7	0	5	5	0.23	4.44	5.94	10
8.	0	12	0	4.90	2.12	2.37	10.8

*ECMO, extracorporeal membrane oxygenator; ICU, intensive care unit; CPB, cardiopulmonary bypass; VIS, vasoactive-inotropic score*.

## Discussion

Our study demonstrated that mild hypothermia with SCP was feasible for aortic arch repair surgery in neonates. During isolated cerebral perfusion, the cerebral oximeter readings were similar during full flow and SCP status. No stroke, seizure, or neurological complications were reported during follow-up. However, renal perfusion was significantly lower in the isolated cerebral perfusion status in mild hypothermia. ~30% of our patients had low urine output for 2 days. All patients recovered favorably. Nevertheless, the safety of a longer SCP for arch repair with mild hypothermia during complex cardiac surgery was questionable.

The optimal perfusion pressure, optimal flow rate, hematocrit, and temperature for SCP in neonate surgery are controversial. Both underperfusion and overperfusion have deleterious effects on neurocognitive function. Overperfusion, either excess pressure or flow, could lead to increased intracranial pressure, cerebral edema, and slow neurocognitive recovery. Underperfusion leads to brain ischemia (16, 17). Mean arterial pressure, either radial artery pressure or femoral artery pressure, had a poor correlation with the bypass flow rate during SCP (18), and real-time measurement of cerebral blood flow, oxygen delivery, oxygen extraction rate, and neuronal stress during cardiac surgery and adjustment of perfusion pressure and flow accordingly are difficult; therefore, non-invasive cerebral oximetry monitoring through NIRS is commonly adopted as an alternative. In adult arch surgery with SCP, the decrease in regional cerebral tissue oxygen saturation between 76 and 86% from baseline had a sensitivity of up to 83% and a specificity of up to 94% in identifying individuals with stroke. In deep hypothermic status, a sustained drop in cerebral rSO2 below 55% correlated with transient neurologic events (19). NIRS is limited to the detection of overperfusion, embolic events, or hypoperfusion in the basilar region. Further measurement of reactive oxygen species, neuronal stress markers, or neuroimaging data is needed to provide more information on cerebral metabolism under SCP.

Randomized controlled trials for neonatal arch surgery have shown that under deep hypothermia, total arrest and selective antegrade cerebral perfusion had similar neurological outcomes (11). However, few studies have compared neurological outcomes of SCP between mild hypothermia and deep hypothermia. Algra et al. (20) demonstrated a correlation of prolonged postoperative recovery with increasing DHCA duration during neonatal arch reconstruction. Cerebral ischemia for 30 min in neonates induces an immediate innate immune response despite cooling the temperature to deep hypothermia (21). Mild hypothermia attenuates the left shift of the hemoglobin dissociation curve, resulting in a better oxygen release in tissue, which might be physiologically better. In a piglet study, cerebral oxygenation and microdialysis findings showed a depletion of the cerebral energy store during circulatory arrest in the DHCA group (22). Although both DHCA and SCP patients in our study recovered favorably with no neurological deficits, SCP patients with mild hypothermia exhibited less cerebral saturation disturbance.

Visceral organ ischemia is not uncommon after neonatal arch surgery and is associated with delayed postoperative recovery. Distal organ ischemia during arch repair is inevitable, and systemic inflammatory responses after ischemia–reperfusion injuries may cause kidney, liver, and intestinal dysfunction (23). During hemorrhagic shock in normothermia, renal blood flow pressure autoregulation is impaired in advance of cerebral blood flow autoregulation (24). A comparison of renal dysfunction incidence by cardiopulmonary bypass core temperature showed that the renal dysfunction incidence was significantly higher in the SCP patients with moderate hypothermia than in those with deep hypothermia (25). Low renal oximetry values during cardiac surgery correlate with the development of acute kidney injury in neonates (26). In our study, the visceral ischemia time for all patients was under 20 min. Nevertheless, two patients had short-term decreased urine output postoperatively. Deep hypothermia in the DHCA patient may have protected the kidney in the absence of any perfusion, as shown by the higher renal rSO2 levels. For complex procedures and longer ischemic duration, a lower core temperature might be more favorable for visceral organ protection.

Our study has some limitations. First, it was an observational study without a randomization design; thus, we could not conclude that mild hypothermia was better for shorter durations in neonatal aortic arch surgery. However, from our experience, mild hypothermia with SCP provided constantly stable intraoperative cerebral oxygenation, with a low inotropic requirement immediately after cardiopulmonary bypass. Moreover, no neurological deficit was noted during hospitalization and follow-up. Second, we included only a few cases, which may have affected the statistical power of analysis. In our institution, we use SCP but in lower core temperature to protect against visceral ischemia in complex aortic arch surgery. Thus, the patient number for SCP with mild hypothermia was limited. However, we hope that the homogeneity in our patients could eliminate unnecessary confounding factors and offer evidence for the feasibility of SCP in neonate arch surgery. Third, we only followed up the patients for neurological outcomes for 6 months and did not undertake brain imaging. Long-term follow-up should be conducted in the future to validate the safety and efficacy of SCP in mild hypothermia for short durations.

## Conclusion

Neonatal arch repair surgery could be performed under mild hypothermia with SCP with low neurological complication risk. However, whether visceral organ ischemia risk is higher with the use of this method warrants further prospective randomized trial study. The SCP parameters need to be individualized and adjusted according to regional saturations.

## Data Availability Statement

The original contributions presented in the study are included in the article/Supplementary Material, further inquiries can be directed to the corresponding author.

## Ethics Statement

The studies involving human participants were reviewed and approved by Naional Taiwan University Hospital. Written informed consent to participate in this study was provided by the participants' legal guardian/next of kin.

## Author Contributions

C-HH: conceptualization, review, and editing manuscript. Y-CW: original draft preparation. H-WC: project administration. S-CH: funding acquisition and supervision. All authors contributed to the article and approved the submitted version.

## Conflict of Interest

The authors declare that the research was conducted in the absence of any commercial or financial relationships that could be construed as a potential conflict of interest.
