# Supplementary material for: Near-Infrared Spectroscopy Assessment of Tissue Oxygenation During Selective Cerebral Perfusion for Neonatal Aortic Arch Reconstruction
Source: Front Med (Lausanne). 2021 Apr 30;8:637257. doi: 10.3389/fmed.2021.637257 (PMC8119641; doi:10.3389/fmed.2021.637257)
Supplement: Supplementary file 1 [file Table_1.DOCX]

**TABLE |** Clinical characteristics of included patients.

|  | GA | BW  (kg) | Age  (day) | Sex | Pre-Operation VIS score | Pre-Operation ventilator | Pre-Operation  MBP  (mmHg) | Pre-Operation  Lactacte (mmole/L) |
| --- | --- | --- | --- | --- | --- | --- | --- | --- |
| 1 | 37+5 | 2.29 | 3 | M | 0 | No | 34.0 | 4.82 |
| 2 | 40+2 | 3.9 | 3 | M | 0 | No | 50.7 | 2.11 |
| 3 | 41+2 | 3.04 | 2 | M | 3.7 | No | 43.0 |  |
| 4 | 39+2 | 3.2 | 4 | M | 0 | Yes | 40.3 | 1.89 |
| 5 | 39+4 | 2.06 | 0 | M | 0 | No | 0.0 | 3.07 |
| 6 | 37+6 | 2.7 | 8 | F | 0 | No | 39.0 | 2.1 |
| 7 | 37+4 | 3.3 | 1 | M | 0 | Yes | 39.7 | 1.77 |
| 8 | 38 | 2.86 | 17 | M | 0 | No | 44.3 | 2.1 |

*GA, gestational age; BW, body weight; MBP, mean blood pressure; VIS, vasoactive-inotropic score.*
